# CD90/Thy-1, a Cancer-Associated Cell Surface Signaling Molecule

**DOI:** 10.3389/fcell.2019.00066

**Published:** 2019-04-26

**Authors:** Chloé Sauzay, Konstantinos Voutetakis, Aristotelis Chatziioannou, Eric Chevet, Tony Avril

**Affiliations:** ^1^INSERM U1242, Proteostasis and Cancer Team, Chemistry Oncogenesis Stress Signaling, Université de Rennes 1, Rennes, France; ^2^Centre Eugène Marquis, Rennes, France; ^3^Institute of Biology, Medicinal Chemistry and Biotechnology, National Hellenic Research Foundation, Athens, Greece; ^4^Department of Biochemistry and Biotechnology, University of Thessaly, Larissa, Greece; ^5^e-NIOS Applications PC, Kallithea-Athens, Greece; ^6^Rennes Brain Cancer Team (REACT), Rennes, France

**Keywords:** THY-1, CD90, cancer, invasion, migration, ER stress, IRE1

## Abstract

CD90 is a membrane GPI-anchored protein with one Ig V-type superfamily domain that was initially described in mouse T cells. Besides the specific expression pattern and functions of CD90 that were described in normal tissues, i.e., neurons, fibroblasts and T cells, increasing evidences are currently highlighting the possible involvement of CD90 in cancer. This review first provides a brief overview on CD90 gene, mRNA and protein features and then describes the established links between CD90 and cancer. Finally, we report newly uncovered functional connections between CD90 and endoplasmic reticulum (ER) stress signaling and discuss their potential impact on cancer development.

## Introduction

**FIGURE 1:**
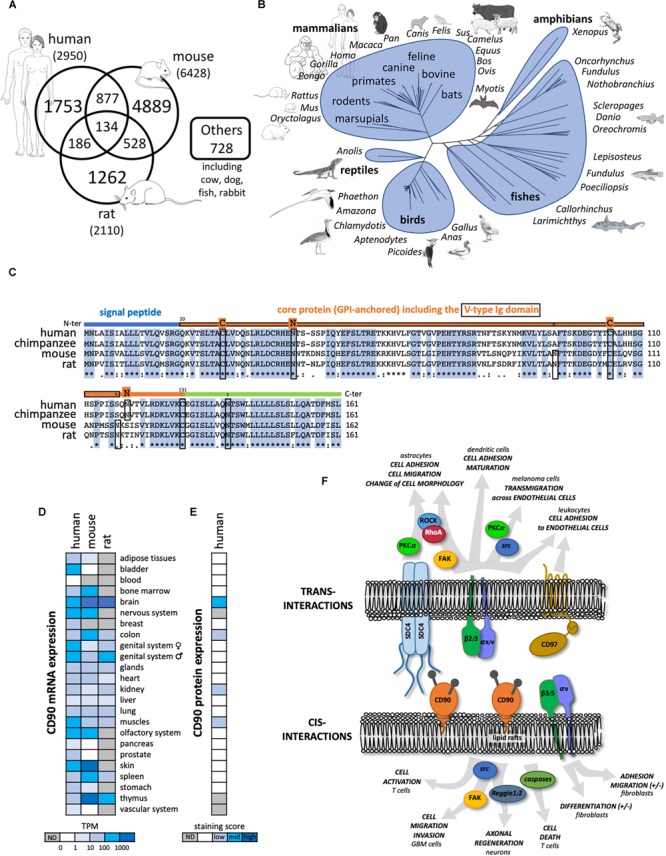
General features of CD90 molecule. **(A)** Number of publications until November 2018 referring to CD90 according to the different species collected in Pubmed (https://www.ncbi.nlm.nih.gov/pubmed). **(B)** Tree representing the evolution of CD90 proteins among vertebrates. **(C)** The CD90 protein sequences from human, chimpanzee, mouse, and rat were aligned showing a highly conserved domains. The main features of the protein including the signal peptide (blue line), the V-type Ig domain (framed orange line), the N-glycosylation sites (n in rodents and N in primates), and the cysteines involved in the di-sulfite bond **(C)** are represented. **(D)** CD90 mRNA expression patterns in normal tissues from human, mouse and rat were analyzed using the EMBL-EBI Expression Atlas (https://www.ebi.ac.uk/gxa/home). **(E)** CD90 protein expression patterns from human normal tissues were tested using the Human Protein Atlas (https://www.proteinatlas.org/). **(F)** CD90 signaling partners and ligands interacting in *cis* and *trans* were summarized including their involvement in different functions and cell types.

Thy-1/CD90 was first identified in 1964 on mouse T lymphocytes (Reif and Allen, 1964a,b) and then on rat thymocytes and neural cells (Barclay et al., 1976). Since then, more than 10, 000 publications refer to CD90 mainly in rodent and human species (Figure 1A). The *CD90* gene is conserved from fish to mammal (vertebrates; Figure 1B), and homologs have been even described in some invertebrates such as squids, tunicates, and worms (Cooper and Mansour, 1989). *CD90* gene organization including promoter region and methylation sites was further described and reviewed in Barclay et al. (1976); Seki et al. (1985); Cooper and Mansour (1989). Importantly, the *CD90* promoter is often considered to be specifically activated in the brain. Consequently, the *CD90* promoter has routinely been used to drive “brain specific” expression of proteins in mice (Feng et al., 2000). The mouse and human CD90 protein are highly similar sharing 66% identity (Figure 1C).

The CD90 protein is a small membrane glycophosphatidylinositol (GPI) anchored protein of 25 to 37 kDa, heavily N-glycosylated on two or three sites in human and mouse, respectively. One third of the CD90 molecular mass is linked to its glycosylation level (Pont, 1987; Haeryfar and Hoskin, 2004). CD90 is composed of a single V-like immunoglobulin domain anchored by a disulfide bond between Cys 28 and Cys 104. CD90 lacks an intracellular domain but is located in the outer leaflet of lipid rafts at the cell plasma membrane allowing signaling functions by *cis*- and *trans*-interactions with G inhibitory proteins, the *Src* family kinase (SFK) members src and c-fyn, and tubulin (Figure 1F; Rege et al., 2006; Avalos et al., 2009; Wandel et al., 2012). Interestingly, similar to what is observed for other GPI-anchored proteins such as CD55 and CD59, CD90 could be shed by specific phospholipases (i.e., PI-PLC or PLC-β) thus allowing cell to cell transfer, however, the physiological relevance of this process remains to be discovered (Haeryfar and Hoskin, 2004).

Common and distinct cellular CD90 expression patterns are observed in mouse and human. CD90 mRNA is highly expressed in nervous and olfactory systems, and skin tissues in both species. However, high CD90 mRNA expression is only found in mouse spleen and thymus (Figure 1D). In the nervous system, CD90 protein expression is observed mainly in neurons but also in some glial cells in vertebrates (Figure 1E). Recently, CD90 has been touted as a stem cell marker in various tissues such as in hematopoietic stem cells used in combination with the CD34 marker but also in hepatic, keratinocyte and mesenchymal stem cells (Kumar et al., 2016). Distinct cellular distributions of CD90 protein expression are observed in mouse (i.e., thymocytes and peripheral T cells) and human (i.e., endothelial cells and smooth muscle cells) (Rege and Hagood, 2006; Barker and Hagood, 2009; Bradley et al., 2009; Leyton and Hagood, 2014). Another important difference between the two species is the existence of two distinct murine isoforms CD90.1 and CD90.2 that differ at the residue 108 (Arg or Gln, respectively) whereas only one isoform is described in human with a histidine at position 108 (Bradley et al., 2009).

Several functions of CD90 have been described so far in physiological and pathological processes (Figure 1F). Most of these functions involve CD90 interactions with ligands such as integrins αv/β3, αx/β2, syndecan-4, CD90 itself, and CD97 (Wandel et al., 2012; Kong et al., 2013; Leyton and Hagood, 2014). CD90 plays a role in cell-cell and cell-matrix interactions, with specific implications in the regulation of axon growth and nerve regeneration, T cell activation and apoptosis, leukocytes and melanoma cell adhesion and migration, fibroblast proliferation and migration in wound healing, inflammation and fibrosis. These functions were already extensively reviewed in Rege and Hagood (2006); Barker and Hagood (2009); Bradley et al. (2009); Leyton and Hagood (2014), and will not be developed further here. Rather, we will focus on CD90 expression and functions in cancers.

## Diverse Roles of CD90 in Cancers

### CD90 Expression in Various Cancer Types

**FIGURE 2:**
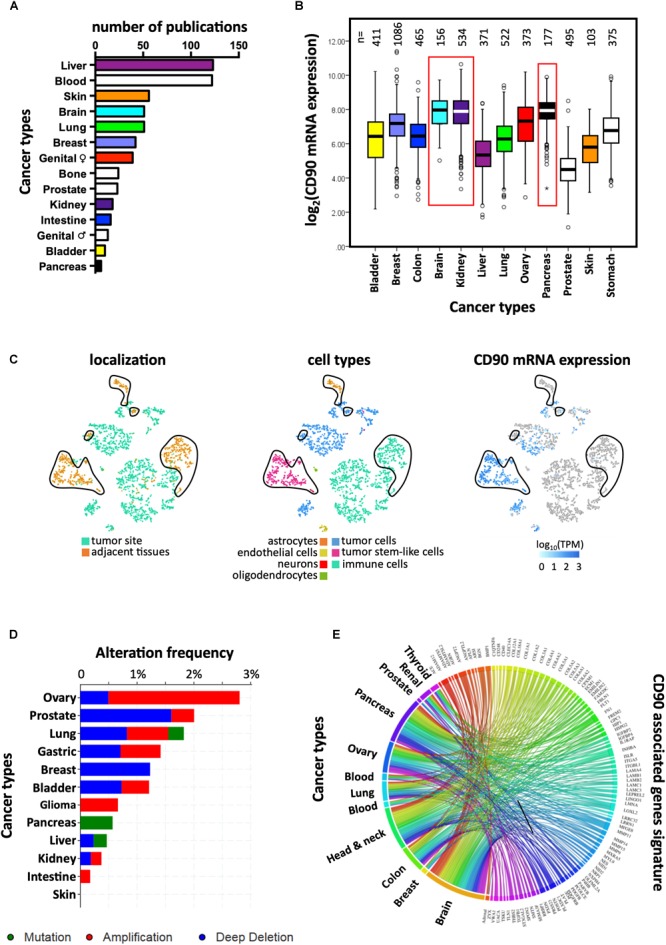
Links between CD90 and cancers. **(A)** Articles reporting CD90 in various human cancer types were collected using Pubmed and their distribution per cancer type is presented. **(B)** CD90 mRNA expression was analyzed among the various cancer types using the TCGA resource. The corresponding number of tumor specimens tested are indicated on the top of the graph. **(C)** CD90 mRNA expression was analyzed in the single cell RNA sequencing dataset from GBM specimens (Darmanis et al., 2017) using the EMBL-EBI Single Cell Expression Atlas (https://www.ebi.ac.uk/gxa/sc/home). Cell localization and cell types are also represented. **(D)** Frequency of *CD90* gene alterations including mutations, gene amplification and deletion was analyzed among the different cancer types. **(E)** CD90 associated gene signature obtained from GBM specimens (Avril et al., 2017a) was tested in others cancer types using CancerMA tool (Feichtinger et al., 2012).

CD90 mRNA and protein expression was reported in several cancer types including liver, myeloid, skin, and brain (Figure 2A). According to The Cancer Genome Atlas (TCGA), CD90 transcripts were predominantly found in brain, kidney, and pancreatic tumors (Figure 2B). CD90 mRNA and protein were detected in glioma/GBM specimens (Wikstrand et al., 1985) and immortalized glioma/GBM cell lines (Kemshead et al., 1982; Seeger et al., 1982; Hurwitz et al., 1983; Wikstrand et al., 1983; Rettig et al., 1986). In the past few years, CD90 has been considered as a human GBM stem cell (GSC) marker (Liu et al., 2006; Kang and Kang, 2007; Tomuleasa et al., 2010; He et al., 2012; Nitta et al., 2015). CD90 is also expressed in GBM-associated stromal cells (GASCs) (Clavreul et al., 2012) and mesenchymal stem cell-like pericytes (Ochs et al., 2013), thereby reflecting GBM cellular heterogeneity. We recently demonstrated that CD90 expression is not only restricted to GBM stem-like cells but is also observed in more differentiated GBM cells (primary adherent lines) and in freshly dissociated GBM specimens (Avril et al., 2017a). Using the recent single-cell RNA sequencing datasets from stem-like and no-stem GBM cells and tumor migrating cells (Darmanis et al., 2017; Cook et al., 2018), we confirm herein that CD90 is expressed in tumor cells from the cancer site but also in migrating tumor cells, tumor-associated endothelial cells, and neighboring neuronal cells (Figure 2C). CD90 expression in kidney cancers is currently controversial. Primary cell lines and tumor stem cells from pediatric Wilms’ tumors and metastatic renal tumors express CD90 (Pode-Shakked et al., 2009; Royer-Pokora et al., 2010; Khan et al., 2016) as observed in renal tumor-associated endothelial cells (Mesri et al., 2013). CD90 is also highly expressed in renal cell carcinoma tumor-initiating cells characterized by CD105 expression (Bussolati et al., 2008; Khan et al., 2016). Nevertheless, CD90 expression could not be found in CSCs derived from patients with clear cell renal cell carcinoma (Galleggiante et al., 2014). The CD90 protein is expressed in almost all the pancreatic adenocarcinoma (PDAC) (*n* = 98) and its metastatic forms tested by tissues microarray (Zhu et al., 2014), not only in tumors cells but also in stromal cells, including fibroblasts, and vascular endothelial cells. In addition, CD90 was extensively studied in liver cancers. Almost no CD90 expressing cells are present in disease-free or in cirrhotic livers, whereas a significantly higher expression is found in hepatocellular carcinoma (HCC) cells (Yang et al., 2008; Sukowati et al., 2013). CD90 protein is also found in esophageal squamous cell carcinomas mainly in primary tumors and immortalized/primary cell lines (Tang et al., 2013). Overexpression of CD90 is also detected in prostate cancer. Indeed immunohistochemical analysis of prostate cancer samples showed distinct and differential overexpression of CD90 in cancer-associated stroma compared with non-cancer tissue stroma (True et al., 2010).

### CD90 Somatic Mutations in Cancers

Mutagenesis is often associated with carcinogenesis. Protein expression or functions could be altered by mutations leading in turn to an oncogenic process. No study has yet reported mutations in the *CD90* gene or CD90-associated related regulatory elements in any of cancer types (Kumar et al., 2016). According to the “Catalog Of Somatic Mutations In Cancer” (COSMIC; *n* = 47210 samples) and cBioPortal for Cancer Genomics (*n* = 52770 samples), the mutation frequency of CD90 in cancer is very low (0.001% with 51 and 54 mutations according to COSMIC and cBioPortal, respectively) (Cerami et al., 2012; Tate et al., 2018). Most of the CD90 mutations are missense (60.8% and 92.6% according to COSMIC and cBioPortal, respectively) and synonymous (35.3%, COSMIC) substitutions mainly found in intestine, lung, and skin cancers (Figure 2D). Only three mutations leading to sequence frameshift were detected in neuroblastoma and renal cancers. In addition, one RGS12/CD90 fusion was detected in a breast invasive lobular carcinoma. Further studies are needed to understand how these mutations could impact on CD90 functions and to clarify the potential roles of these mutations in cancers.

### CD90 as a Cancer Stem Cell Marker

The cancer stem cell (CSC) concept has been proposed four decades ago, and states that tumor development is driven by a specialized cell subset, characterized by self-renewing, multi-potent, and tumor-initiating properties (Batlle and Clevers, 2017). In recent years, the role of CD90 was extensively studied in CSCs (Shaikh et al., 2016). The ability to form tumors *in vivo* in immunodeficient mice is considered to be one of the most important properties of CSCs. CD90+ tumor cells, considered as CSCs, from several cancers, i.e., HCC (Yang et al., 2008), gastric cancers (Jiang et al., 2012) and esophageal squamous cell carcinomas (Tang et al., 2013) were reported to form tumors in immunodeficient mice after injection of a very small amount of cells in contrast to CD90 negative counterparts. Another important feature of CSCs is their ability to grow *in vitro* as spheroids in serum-free medium. This feature has been recapitulated using CD90 expressing cells obtained from esophageal squamous cell carcinomas (Tang et al., 2013), gastric cancers (Jiang et al., 2012), gliomas (Kang and Kang, 2007; He et al., 2012), and lung carcinomas (Wang et al., 2013). Taken together, these studies identify CD90 as a potential CSC marker in many types of cancers. However, we have recently demonstrated in GBM that CD90 is not only a stem marker, as its expression is also observed in more differentiated GBM cells (Avril et al., 2017a).

### CD90 as a Tumor Suppressive Molecule or a Prognostic Marker in Cancers

The prognostic role of CD90 is dependent on the cancer type. In GBM patients, high expression of CD90 in tumor specimens is associated with invasive features as demonstrated by imaging techniques (Avril et al., 2017a). These imaging features were previously linked to shorter patients’ survival (Colen et al., 2014). Therefore, we proposed that CD90 expression could represent a novel stratification tool for screening patients with highly invasive tumors that could be treated with dasatinib, a SFK inhibitor. Moreover, dasatinib could not only impair the adhesion/migration of CD90^high^ differentiated tumor cells but also the proliferation of CD90^high^ GSCs, thereby increasing its therapeutic potential in CD90^high^ tumors (Avril et al., 2017a). In hepatoblastoma, increased expression of CD90 is significantly correlated with advanced stages of the disease, poor response to treatment and lower overall survival (Bahnassy et al., 2015). CD90 overexpression was also identified as a poor prognostic marker in acute myeloid leukemia (Buccisano et al., 2004) and HCC (Lu et al., 2011). In contrast, CD90 was also shown to exert tumor suppressor functions in several others cancers, as its downregulation is associated with poor prognosis, disease progression in ovarian adenocarcinoma (Gabra et al., 1996; Abeysinghe et al., 2003), neuroblastoma (Fiegel et al., 2008) and nasopharyngeal carcinoma predominantly observed in metastatic tumor cells in invaded lymph nodes (Lung et al., 2005). CD90 inactivation is found associated with hypermethylation of the *CD90* gene promoter in CD90 negative nasopharyngeal carcinoma cell lines. Furthermore, induction of CD90 expression in nasopharyngeal carcinoma and ovarian cell lines leads to inhibition of tumor growth *in vitro* and *in vivo*, respectively (Abeysinghe et al., 2003; Lung et al., 2005). Overall, these observations illustrate the ambivalence of CD90 functions with either pro- or anti-tumoral properties depending on the cancer type.

### CD90 Regulates Tumor Migration and Metastasis

Tumor invasion/migration is one of cancer hallmarks that drives to tumor dissemination leading to disease aggravation. To spread within the tissues, tumor cells use migration mechanisms that are similar to those occurring in physiological processes, including mesenchymal, amoeboid single migration or collective movements, depending on the cancer type (Friedl and Wolf, 2003; Odenthal et al., 2016). Recent studies demonstrated high invasive and metastatic capacities of CD90 expressing cells in several cancers. Indeed, NOD/SCID mice implanted subcutaneously with HCC tumor cells expressing both CD90 and CXCR4 developed distal metastatic tumors (Zhu et al., 2015). Similarly, the high metastatic capacity of CD90 and EpCAM expressing cells from primary HCC has also been observed after subcutaneously injection in immune-deficient mice (Yamashita et al., 2013). These cells have the capacity to invade surrounding tissues, to form spheroids *in vitro* and to exhibit high expression of TWIST1 and TWIST2, two important transcription factors involved in activation of Epithelial to Mesenchymal Transition (EMT) process. In addition, the presence of CD90 positive cells in HCC patients was also associated with a higher incidence of distant organ metastasis (usually occurring in one third of HCC patients) including lung, bone and adrenal gland; within 2 years after surgery (Yamashita et al., 2013). In a recent study, we demonstrated the critical role of CD90 in GBM migration/invasion mainly through the activation of SRC signaling. The same study demonstrated CD90 association with a cell adhesion/migration gene signature and with multifocal/multicentric MRI features in GBM patients. Importantly, this adhesion/migration profile is also found in other CD90-expressing tumors such as colonic, pancreatic and ovarian cancers (Figure 2E). Moreover, orthotopic xenografts revealed that CD90 expression induced invasive phenotypes *in vivo* that could be inhibited by dasatinib (Avril et al., 2017a). In melanoma, CD90 expressing endothelial cells are mainly associated with highly metastatic tumors (Ohga et al., 2012). CD90 also mediates adhesion of melanoma cells to activated human endothelial cells via its interaction with the αv/β3 integrin on the tumor cells *in vitro* (Saalbach et al., 2005). Furthermore, expression of αv/β3 integrin (CD51/CD61), one of the CD90 ligands, in melanoma cells is associated with tumor progression and metastases formation (Ohga et al., 2012). As VEGF and TNFα induce the expression of CD90 in endothelial cells, it has been shown that mice lacking CD90 showed markedly diminished experimental lung metastasis after injection of B16/F10 melanoma cells compared to wild-type controls (Schubert et al., 2013). Interestingly, a subpopulation of breast cancer cell line MDA-MB-231 expressing CD90 and CD105, exhibits mesenchymal stem cell-like characteristics such as high migratory capacity as compared to the parental and CD90/CD105 negative cells (Wang et al., 2015). Remarkably, in breast tumor specimens, tumor cells that express both CD90 and CD44 are confined to the periphery of the tumor, representing the tumor invasive front (Donnenberg et al., 2010).

## Emerging Roles of the Unfolded Protein Response in Cancers: a Novel Link Between CD90 and Er Stress?

During tumor invasion/migration, dramatic changes occur in cells present within the compact tumor core to become single migrating cells, these transformations are described as the EMT throughout which, cells lose their cell-cell junctions, change their morphology and modify their functions leading to cell trans/de-differentiation (Friedl and Wolf, 2003; Thiery et al., 2009). Tumor development and aggressiveness including invasion and EMT were recently linked to Endoplasmic Reticulum (ER) stress signaling (Dejeans et al., 2015; Urra et al., 2016), a topic that we will further document in the next sections. Since both the signaling response triggered to cope with ER stress [also named Unfolded Protein Response (UPR)] and CD90 expression promote tumor migration, we hypothesize that CD90 and the UPR could be somehow functionally linked to control tumor cell invasive.

### An Overview on UPR and Its Sensors

**FIGURE 3:**
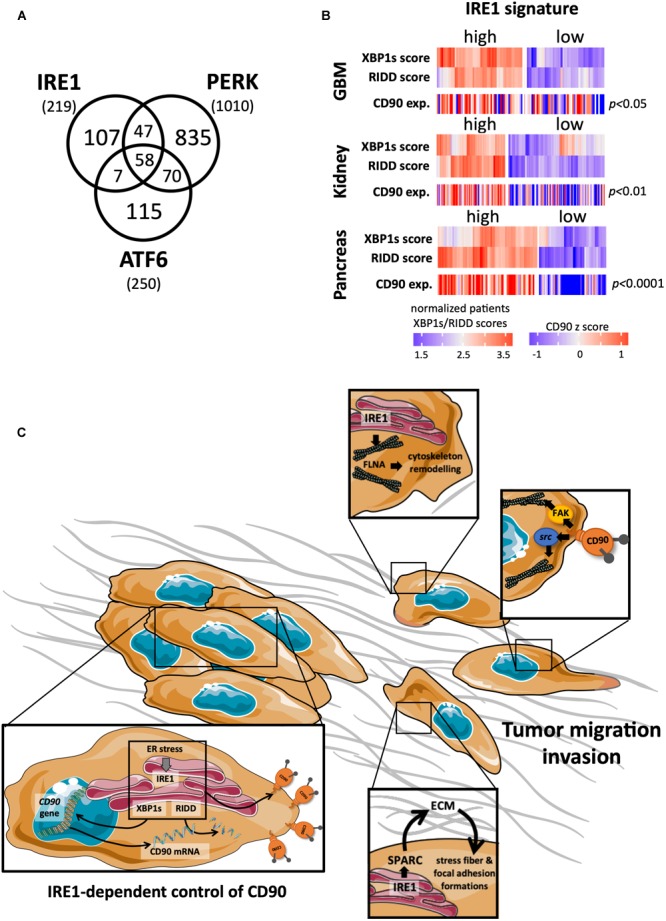
Links between CD90 and IRE1 activity in cancers. **(A)** Articles describing a role for the ER stress sensors IRE1, PERK, and ATF6 in human cancer disease were collected using Pubmed and their distribution represented per sensor type. **(B)** CD90 mRNA expression was analyzed according to IRE1 activity from GBM, kidney and pancreas cancers (TCGA resources). Cancer patients were classified in regard to IRE1 and XBP1s/RIDD activities as described in Lhomond et al. (2018). **(C)** A schematic representation of the possible CD90 regulation by IRE1 is presented.

Despite an elaborate network of chaperones, foldases and proteins involved in the quality control of newly synthesized proteins, the ER capacity for protein synthesis and folding can be overwhelmed upon various physiological and pathological conditions, causing an accumulation on misfolded proteins into the ER and a cellular stress called ER stress. To cope with ER stress, cells activate an adaptive signaling pathway named the UPR. The UPR activates a cascade of signals leading to the attenuation of mRNA translation and to the transcriptional increase of genes whose products are involved in ER protein folding, ER protein quality control, ER-associated degradation and protein secretion. If ER stress persists, the UPR signaling shifts from adaptive to apoptotic signals thus leading to the tumor cell death (Chevet et al., 2015; Avril et al., 2017b). The UPR activation relies on 3 ER resident proteins/sensors ATF6α, IRE1α (for Inositol-Requiring Enzyme 1 alpha; referred to as IRE1 hereafter) and PERK. Initially, it was postulated that the activation of these three sensors was controlled by the ER resident chaperone GRP78/BIP and misfolded proteins themselves, thoroughly reviewed in Chevet et al. (2015); Urra et al. (2016); Avril et al. (2017b). More recently, novel mechanisms and actors of ER stress sensors activation have been described (Rojas-Rivera et al., 2018) such as the involvement of the ATP binding pocket of BIP (Carrara et al., 2015; Kopp et al., 2018); the involvement of the chaperones ERDJ4 (Amin-Wetzel et al., 2017) and HSP47 (Sepulveda et al., 2018), the ER oxidoreductase PDIA6 (Eletto et al., 2014; Groenendyk et al., 2014), and the other protein disulfide isomerase PDIA5 (Higa et al., 2014). During cancer development, tumor cells are exposed to intrinsic challenges (related to activation of their oncogenic program or aneuploidy) and to extrinsic stresses (related to nutrient and oxygen deprivation, but also anti-cancer treatments as such irradiation or chemotherapy), which lead to an altered balance between protein folding demand and the capacity of transforming cells to cope with this, thus driving ER stress (Chevet et al., 2015; Urra et al., 2016; Avril et al., 2017b; Galmiche et al., 2017). UPR sensors have been largely studied in regard to cancer diseases (Figure 3A). A strong involvement of one of these UPR sensors, IRE1, has been recently reported in GBM biology (Drogat et al., 2007; Auf et al., 2010; Dejeans et al., 2012; Pluquet et al., 2013; Jabouille et al., 2015; Obacz et al., 2017; Lhomond et al., 2018) and will be now presented.

### IRE1 Activation and Down-Stream Signaling Pathways

IRE1 dimerizes/oligomerizes upon ER stress. This leads to its *trans-*autophosphorylation which in turn induces a conformational change to activate IRE1 endoribonuclease domain (RNase) (Chevet et al., 2015). The activation of IRE1 RNase triggers two distinct signaling pathways that lead to (i) the non-conventional splicing of the XBP1 mRNA into a novel mRNA encoding a transcription factor XBP1s; and (ii) the degradation of RNA (also called RIDD for regulated IRE1-dependent decay of RNA). XBP1s is a transcription factor that controls the expression of genes involved in protein folding, secretion, ERAD, and lipid synthesis (Chevet et al., 2015). On the other hand, RIDD targets mRNA, ribosomal RNA and microRNAs. Importantly, the selectivity of IRE1 RNase activity is highly dependent on its oligomerization state; a concept still debated as to its specificity and application (Chevet et al., 2015). IRE1 activation has also been shown to lead to c-Jun N-terminal protein kinase (JNK) phosphorylation through either the recruitment of TRAF2 (Urano et al., 2000) or the cleavage of miR17 (Lerner et al., 2012).

### A Key Role of IRE1 in GBM Pathology

One of the central hallmarks of GBM is the diffuse infiltration of tumor cells into the cerebral neighboring parenchyma (Louis et al., 2007), making a complete tumor resection almost impossible (Zhong et al., 2010; Vehlow and Cordes, 2013). Interestingly, inhibition of IRE1 reduces GBM growth *in vivo* (Drogat et al., 2007; Auf et al., 2010) but alters tumor cell migration/invasion properties (Dejeans et al., 2012; Jabouille et al., 2015), acting for instance on SPARC expression, a molecule associated with the extracellular matrix (Dejeans et al., 2012). Furthermore IRE1, through its dual XBP1s and RIDD activities, exerts antagonistic effects on GBM aggressiveness influencing both tumor invasion, neo-angiogenesis and inflammation (Lhomond et al., 2018). Remarkably, GBM patients bearing tumors characterized by high IRE1 activity (and more precisely high XBP1s) exhibit a worse prognosis and display increased immune infiltration, angiogenesis and migration markers. This also opens interesting perspectives of connections between IRE1 activity and the growth and the invasion of GBM cells; however, further studies are required to understand how IRE1 downstream signaling impacts on these features.

### Possible Links Between IRE1 and CD90 in Controlling GBM Cell Migration/Invasion

As our two recent studies independently demonstrated that CD90 and IRE1 could regulate GBM migration/invasion features, specific functional connections between these two proteins were considered herein. For instance, when tumors developed in mouse brain in our orthotopic mouse model, similar features of tumor infiltration, i.e., small but highly invasive tumors have been observed in CD90 expressing (Avril et al., 2017a) and IRE1 defective (Dominant Negative, DN; Auf et al., 2010) U87 cells. Due to its RNase activity, one could speculate that a functional regulatory link might exist between IRE1 and CD90 which in turn could therefore impact on GBM CD90-dependent migration. Ongoing studies from our laboratory aim at directly investigating the link between CD90 and IRE1 activity. Preliminary data indicate that ER stress inducers (such as tunicamycin, thapsigargin, and dithiothreitol) decrease the expression of cell surface CD90 in GBM cells. Intriguingly, transient expression of IRE1 defective form (DN) in U251 cells also decreased membrane CD90 expression, underlining a complex regulatory mechanism occurring between ER stress sensors (including IRE1) and CD90. Importantly, applying our recent classification of GBM patients according to IRE1 gene signature on the GBM TCGA cohort, we observed that tumors with high IRE1 activity expressed higher levels CD90 mRNA than tumors exhibiting low IRE1 activity. Importantly, this could be applied to others cancer types including renal and pancreas (Figure 3B). Furthermore, a CD90 associated gene signature described in Avril et al. (2017a) was also associated with IRE1 activity. Overall, these observations highlight the potential effect of IRE activity on CD90 expression and its potential role in functions linked to tumor migration/invasion. Interestingly, IRE1 has already been associated with molecules involved in cell migration, i.e., by controlling SPARC expression (Dejeans et al., 2012) and interacting directly with filamin A (Urra et al., 2018). Further functional and molecular studies are needed to better understand the connections between CD90 and IRE1 in cancer development, and in particular migration and invasion (Figure 3C).

## Conclusion

Increasing evidence supports the importance of CD90 in cancer development. CD90 has been mainly considered as a useful CSC marker in various cancer types such as kidney, brain, and liver. However, even despite the absence of intracellular domain, CD90 is also able to transmit intracellular signals that lead to the activation of tumor cell migration/invasion program in liver and lung cancers, in GBM and in melanoma. In contrast, CD90 is described as a tumor suppressor molecule in nasopharyngeal carcinoma. Although further studies are required to clearly demonstrate the connections between ER stress signaling and CD90 expression, initial transcriptome analyses from cancer patients indicate that CD90 expression appears to be dependent on the activation of the UPR, a key event in various oncological clinical settings including brain, kidney, and pancreatic cancers. These different elements underline the complexity of CD90 functions in cancer, depending on both the cellular context and on the tumor microenvironment. Future studies will lead to the better understanding of CD90 regulation and functions adding to the information already available such as the CD90 mutation spectrum as seen in COSMIC/cBioPortal; the IRE1-controlled CD90 tumor expression and functions with a clarification of the involvement of the IRE1 downstream signaling pathways XBP1s and/or RIDD branches; and the relevance of cleaved CD90 released in the tumor microenvironment.

## Author Contributions

CS wrote the sections of the manuscript. KV and AC organized the database and performed the statistical analysis. EC and TA contributed to conception and design of the review. TA wrote the first draft of the manuscript. All authors contributed to manuscript revision, read, and approved the submitted version.

## Conflict of Interest Statement

The authors declare that the research was conducted in the absence of any commercial or financial relationships that could be construed as a potential conflict of interest.
